# Inflammatory modulation of the associations between prenatal maternal depression and neonatal brain

**DOI:** 10.1038/s41386-020-0774-0

**Published:** 2020-07-20

**Authors:** Yonghui Wu, Han Zhang, Changqing Wang, Birit F. P. Broekman, Yap-Seng Chong, Lynette P. Shek, Peter D. Gluckman, Michael J. Meaney, Marielle V. Fortier, Anqi Qiu

**Affiliations:** 1 Department of Biomedical Engineering, National University of Singapore, Singapore, Singapore; 2 Singapore Institute for Clinical Sciences, Singapore, Singapore; 3 Department of Obstetrics & Gynaecology, Yong Loo Lin School of Medicine, National University of Singapore, National University Health System, Singapore, Singapore; 4 Department of Pediatrics, Khoo Teck Puat – National University Children’s Medical Institute, National University of Singapore, Singapore, Singapore; 5 Ludmer Centre for Neuroinformatics and Mental Health, Department of Psychiatry, Faculty of Medicine, McGill University, Montreal, Canada; 6 Department of Diagnostic and Interventional Imaging, KK Women’s and Children’s Hospital, Singapore, Singapore; 7 The N.1 Institute for Health, National University of Singapore, Singapore, Singapore

**Keywords:** Genetics of the nervous system, Translational research, Human behaviour, Development of the nervous system

## Abstract

Inflammatory signaling has a role in sensing intrauterine environment, which may be moderators in altering fetal brain development upon maternal environment. This study integrated cytokine transcriptome of post-mortem fetal brains, neonatal brain imaging and genetic variants (*n* = 161) to examine whether cytokines are candidates for modulating the relationship between prenatal maternal depression and fetal brain development. This study obtained the transcriptome data of 208 cytokine genes in 12 fetal brain regions from the BrainSpan database. We also included 161 mother–child dyads with prenatal maternal depressive symptoms assessed at 26 weeks of gestation, cytokine genotype data extracted from umbilical cord specimens, and neonatal brain images from a longitudinal prospective birth cohort. We revealed that 22 cytokine genes are expressed in specific brain regions in utero, whose variants have roles in modulating the effects of the prenatal environment on the accelerated fetal development of the hippocampus, auditory, parietal, orbitofrontal, and dorsal prefrontal cortex. Neonates high in the genetic expression score (GES) of TNFRSF19 and IL17RB showed a larger right hippocampal volume, high in the GES of BMPR1B showed the thicker thickness of the sensorimotor cortex, and high in the GES of IL1RAP and CXCR4 demonstrated the thicker thickness of the dorsal and orbital prefrontal cortex in relation with greater prenatal maternal depressive symptoms. Our findings suggest that in humans, the cytokine genes are expressed in a brain region-specific manner in utero and may have potential roles in modulating the fetal development of the corresponding brain regions in response to the maternal environment.

## Introduction

Emerging evidence arising from observational studies in humans reveals that fetal exposure to maternal depression during pregnancy has a long-term impact on the brain development of the offspring, especially brain regions related to affective disorders [1–3]. Exposure to prenatal maternal depression predicts significant alterations of the amygdala microstructure, volume, and its functional connectivity with the orbitofrontal and superior temporal cortices in infancy and childhood [4–8]. Moreover, the altered functional connectivity between the amygdala and prefrontal cortex is observed in infants and children exposed to greater prenatal maternal depressive symptoms [9]. Beyond the amygdala, thickness in the inferior frontal and temporal cortex and microstructure in the white matter of the inferior frontal area are correlated with prenatal maternal depressive symptoms in children aged between 2.6 and 5.1 years old [7]. These brain regions are implicated in psychiatric disorders such as depression and anxiety [10]. These findings suggest that maternal depressive symptoms during pregnancy appear to affect brain development in a manner that influences the vulnerability to affective psychopathology.

Inflammatory processes contribute to the pathogenesis of major depressive disorder (MDD). Cytokines, inflammatory signaling proteins, sense the intrauterine environment, and are plausible candidates for facilitating the maternal–fetal transmission of vulnerability to offspring brain development with implications of affective psychopathology [11–13]. Pro-inflammatory cytokine levels are elevated due to maternal depression during pregnancy [14]. Cytokines can cross the placenta, reach the fetus, and their high levels persist in offspring long after birth [15]. Evidence from animal research suggests that maternal cytokines, such as interleukin IL-1β, IL-8, IL-6, IL-10, and tumor necrosis factor-alpha (TNF-α), interfere with neurogenesis, neural migration, and synaptogenesis in the prefrontal cortex, hippocampus, and amygdala of the offspring [16, 17]. In humans, maternal IL-6 levels during pregnancy are associated with the neonatal amygdala volume and functional connectivity with the hippocampus and sensory cortex that, in turn, predict impulse control of 2-year-old children [11, 18].

Based on above, we hypothesized that inflammatory cytokines may be critical moderators in altering the fetal brain development upon the maternal environment, particularly in the brain regions related to affective psychopathology, such as the amygdala, hippocampus, frontal and sensory cortex [15, 16]. Although this hypothesis is consistent with existing findings, there is to date no direct analysis of cytokines as moderators of the association between maternal mental health and in utero brain development.

**Fig. 1:**
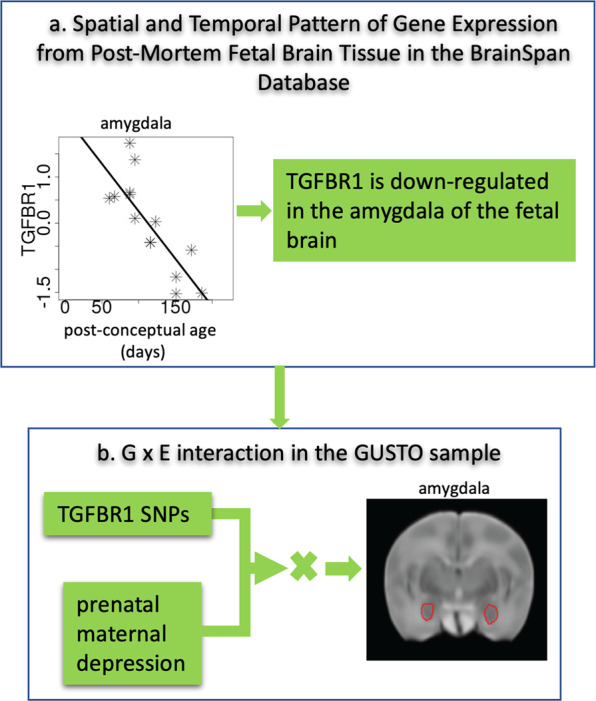
Illustration of a novel bioinformatic approach to examine gene and environment interactions between cytokine genes and prenatal maternal depression on neonatal brain morphology. We used the amygdala as an example. **a** We identified the spatial and temporal pattern of genes expressed in the amygdala in utero (e.g., TGFBR1) based on the transcriptome data of the fetal brain in the BrainSpan database. **b** We extracted the variants of the genes identified in **a** (e.g., the genetic variants of TGFBR1 SNPs). We then employed a gene set-based mixed effect model for gene–environment interaction (MixGE) on the neonatal amygdala morphology.

This study examined this hypothesis based on the maternal measures, neonatal brain images, and cytokine genetic variants of mother–child dyads (*n* = 161) from a prospective longitudinal birth cohort study, the Growing Up in Singapore Towards healthy Outcomes (GUSTO). Genetic variation in a population is commonly studied through the analysis of single nucleotide polymorphisms (SNPs), which have no information about where and when genes are expressed (i.e., upregulated or downregulated). To overcome this limitation of SNPs, this study employed the transcriptomic data of cytokine and chemokine genes obtained from the post-mortem fetal brain tissues available at the BrainSpan database and examined which cytokine or chemokine genes are expressed in utero in a specific brain region (see illustration in Fig. 1). A transcriptome represents that a small percentage of the genetic code that is transcribed into RNA molecules and captures a level of complexity that the simple genotyping sequence does not. Studying transcriptomes allows us to determine when and where genes are turned on or off in various brain regions [19]. The number of transcripts can be quantified to get some idea of the amount of gene activity or expression in a brain region [19]. Hence, by considering the transcriptome, it is possible to generate a comprehensive picture of what genes are active at various stages of development. There is a growing trend to prioritize the genetic variants of candidate genes with a clear functional mechanism determined by transcriptome data, which has been used in studying genetic functional mechanisms underlying brain diseases [20]. In this study, only the genetic variants of the cytokine or chemokine genes, that are upregulated or downregulated in utero in a specific brain region, were used to examine their moderation role in the relationship between prenatal maternal depressive symptoms and the neonatal brain morphology.

Given the fact that SNPs in combination as a polygenic risk score well predict the risk for depression [21] and moderate the influence of childhood maltreatment on depressive symptoms [22], we then employed an advanced gene set-based mixed effect model for gene–environment interaction (MixGE) [23] to test for interactive effects between neonatal genetic variants of each cytokine gene and prenatal maternal depressive symptoms on neonatal brain morphology. The MixGE takes a set of SNPs and examines not only accumulative but also heterogeneous interactive effects of these individual SNPs with prenatal maternal depressive symptoms on neonatal brain morphology. The MixGE model can also detect both rare genetic variants and common genetic variants with small effect sizes by accumulating their effects within the gene set, and by greatly reducing the number of independent statistical tests hence a less stringent correction for multiple comparisons is required [24]. In this study, we applied the MixGE model to individual subcortical volumes and thickness of cortical regions that had the transcriptome data from the post-mortem fetal brain tissues. We employed thickness for the cortex since it is considered as a sensitive measure of the cortical morphology in early life [25, 26].

This study reflects a very novel strategy for the integrative analysis of gene expression and genotypes on the examination of gene–environment interdependence and identifies candidate cytokine genes that might modulate the influence of prenatal maternal depressive symptoms on brain development in utero. This analysis is therefore a novel and important step towards elucidating a potential inflammatory pathway by which prenatal maternal depression influences fetal brain development in the offspring.

## Material and methods

### Cytokine and chemokine genes are expressed in the post-mortem fetal brain

We aimed to identify cytokine and chemokine genes that are up (or down)-regulated in specific brain regions in utero. Figure 1a illustrates the analysis flow of the gene expression. We employed the gene database from PubMed (https://www.ncbi.nlm.nih.gov/pmc/) and used keywords “interleukin receptors”, “TNF receptors”, “TGF beta receptors”, “IFN receptors”, or “Chemokine receptors” to search and select cytokine and chemokine genes, resulting in 237 genes.

Next, we extracted the expression levels of the 237 cytokine and chemokine genes of 12 regions from the fetal brain samples aged at 8–26 post-conceptional weeks (pcw) (8 males and 6 females) that are available in the BrainSpan Atlas (Allen Institute for Brain Science; http://www.brainspan.org). This study included all the 12 brain regions available in the fetal brain samples, such as the amygdala, hippocampus, primary auditory, visual, motor, and sensory cortex, superior temporal cortex, inferior parietal cortex, inferior temporal cortex, anterior cingulate, ventrolateral frontal cortex, dorsolateral frontal cortex, and orbitofrontal cortex. Twenty-nine genes of the 237 cytokine and chemokine genes did not have the expression data in this age range, resulting in 208 genes included in this study. Table S1 in the Supplementary Material lists these 208 genes.

Last, we employed robust linear regression to examine the upregulation or downregulation pattern of the cytokine and chemokine gene expression during the fetal stage [27]. In the regression model, the expression level of an individual cytokine or chemokine gene in each brain region was a dependent variable and the post-conceptional age was an independent variable. False discovery rate was used to correct for multiple comparisons across the 208 genes and the 12 brain regions (corrected *p*-value < 0.01).

### Subjects

The Growing Up in Singapore Towards healthy Outcomes (GUSTO) cohort study was approved by the National Healthcare Group Domain Specific Review Board (NHG DSRB) and the Sing Health Centralized Institutional Review Board (CIRB). Written informed consent was obtained from mothers.

Mother–child dyads who participated in this imaging genetic study were part of the GUSTO cohort whose study design and protocol were described in [28]. The GUSTO screened pregnant Asian women when attending the first-trimester antenatal ultrasound scan clinic at the National University Hospital (NUH) and KK Women’s and Children’s Hospital (KKH) in Singapore. Pregnant women who were Singapore citizens or Permanent Residents of Chinese, Malay or Indian ethnic background, and had no prior history of mental illness, diabetes, other major illnesses, and substance use were recruited. Socioeconomic status represented by maternal education, ethnicity, and age were extracted from survey questionnaires conducted as part of a scheduled appointment during the 26th week of pregnancy. Birth outcomes, including gestational age, birth weight, Appearance, Pulse, Grimace, Activity, and Respiration (APGAR) score, and sex were obtained from the hospital record.

This imaging study only included children with maternal reports on depression scales and without pregnancy complications, and with gestational age, ≥34 weeks, birth weight ≥ 2 kg, and a 5-min APGAR score ≥ 9 to avoid potential effects of pregnancy and birth complications on brain development. Of the 184 mother–offspring dyads with neonatal neuroimaging data, those without genotype data (*n* = 9), prenatal maternal depression scores (*n* = 10), demographic information (*n* = 1) or APGAR score < 9 (*n* = 3) were removed, leaving 161 mother–offspring dyads in this study. Figure S1 in the Supplementary Material shows the flow chart of the subject selection for this study.

### Maternal depressive symptoms

In GUSTO, the Edinburgh Postnatal Depression Scale (EPDS) questionnaire [29] was administered to mothers at 26 weeks of pregnancy to quantify depressive symptomatology. The EPDS is a widely used 10-item self-report scale designed as a screening instrument for postnatal depression and has been well validated for use in prenatal depression. Previous studies confirmed the bi-dimensional factor structure of the EPDS in pregnancy (anxiety and depression, correlation coefficient = 0.538 in the GUSTO sample) and the stability of this factor structure of the instrument across the perinatal period [30–32]. In the EPDS, each item is scored on a four-point scale (0–3), and items three and five-ten are reverse scored. The reliability of the EPDS score was 0.82 assessed using Cronbach’s analysis for our cohort. A higher score indicates a higher intensity of depressive symptoms.

### MRI acquisition and analysis

In GUSTO, neonates at 5–14 days of age underwent axial fast spin-echo T2-weighted MRI scanning (Repetition time (TR) = 3500 ms; Echo Time (TE) = 110 ms; field of view (FOV) = 256 mm × 256 mm; matrix size = 256 × 256; 50 axial slices with 2.0 mm thickness) using a 1.5-Tesla GE scanner at the Department of Diagnostic and Interventional Imaging of the KK Women’s and Children’s Hospital. Detailed acquisition and image quality check procedures were previously reported [33, 34].

Within individual subjects, a Markov random field model (MRF) was used to automatically delineate the 12 brain structures from the neonatal T2-weighted MRI data. These 12 brain regions were chosen due to the availability of their transcriptome data in post-mortem fetal brain tissues. The segmentation accuracy rates of the automatic segmentation against the manual segmentation were reported in previous publications [33–35].

### SNP genotyping

The GUSTO study extracted genomic DNA from frozen umbilical cord specimens for neonates per standard procedures. The samples were then genotyped on both Illumina OmniExpress and Exome arrays. Data were processed in GenomeStudio Genotyping Module™. Genotyping calls were made by the GenCall software that provides the GenCall score of each SNP probe and the call rate of each sample to rank and filter failed genotypes [36]. The genotypes with a GenCall score of <0.15 were not assigned genotypes [36].

This study employed IMPUTE2 to impute the genotype data based on the reference of 1000 Genomes [37]. These SNPs selected in this study did not deviate from Hardy–Weinberg proportions after correction for multiple comparisons. Table S1 in the Supplementary Material lists the number of SNPs of each cytokine gene included in this study.

### Statistical analysis

Figure 1b illustrates the flow of data analysis. We examined interactive effects between genetic variants of the cytokine and chemokine genes, that are expressed in individual brain regions in utero, and prenatal maternal depressive symptoms on their brain morphology (cortical thickness or subcortical volume) at birth using a gene set-based mixed effect model for gene–environment interaction (MixGE; https://bieqa.github.io/imaginggenetics.html) [23, 38]. The MixGE model was in the form of

$${\mathrm{Y}} = Z\beta + {\mathrm{diag}}\left( E \right)G\pi + {\mathrm{diag}}\left( E \right)G\delta ,$$

where *Z* = [*X*, *E*, *G*], where *X* consisted of sex, age on the MRI visit day, total brain volume, maternal education, and ethnicity as covariates. Here, we used maternal education, instead of household income, to represent socioeconomic status since it was highly correlated with household income and a stable measure for SES across time.*Y* was the brain measure. *E* was the score of prenatal maternal depressive symptoms, and *G* represented the SNPs of individual genes. diag(*E*)*G* therefore represented gene–environment interaction (*G* × *E*). *π* was a fixed effect of *G* × *E*. *δ* were random effects of *G* × *E*. *π* captured the accumulative *G* × *E* effects, whereas *δ* captured the heterogeneous *G* × *E* effects among all the SNPs. The same statistical analysis was applied to individual brain morphological measures and genes that were expressed in this brain region during gestation.

When the interaction was significant, we followed post hoc analysis where a genetic expression score [39] was calculated for individuals by summing the number of minor alleles across the SNPs of the gene that were highly correlated with its expression level according to the existing expression quantitative trait loci (eQTL) database (https://gtexportal.org/). A higher GES indicates a higher expression level of the gene. Simple slope analysis was then used to examine the associations between prenatal maternal depressive symptoms and the brain measure in the high GES group (+1 SD above the mean of GES) and low GES group (−1 SD below the mean of GES). The same set of covariates, such as sex, age on the MRI visit day, total brain volume, maternal education, and ethnicity, were included in the post hoc analysis.

## Results

### Cytokine genes expressed in specific post-mortem brain regions in utero

**Fig. 2:**
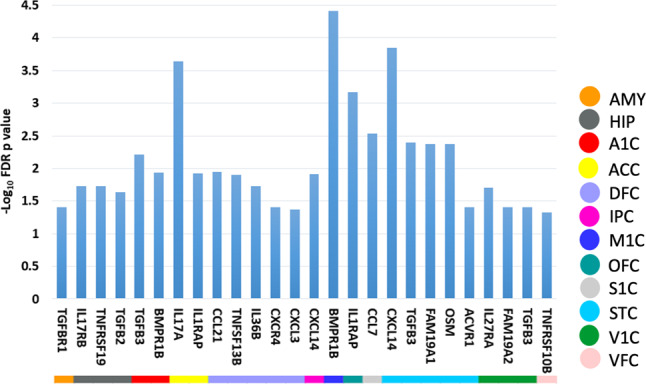
The statistical map of the cytokine genes significantly associated with the post-conceptual age in specific fetal brain regions. The color of solid circles indicates the anatomical annotation of the brain regions. AMY amygdala, HIP hippocampus, A1C primary auditory cortex, ACC anterior cingulate cortex, DFC dorsolateral frontal cortex, IPC inferior parietal cortex, ITC inferolateral temporal cortex, M1C primary motor cortex, OFC orbitofrontal cortex, S1C primary sensory cortex, STC superior temporal cortex, V1C primary visual cortex, VFC ventrolateral frontal cortex.

Robust regression identified 22 cytokine genes whose expression levels in specific brain regions were significantly associated with the post-conceptual age. Figure 2 summarizes the significance of such a relationship in specific fetal brain regions. Figure S2 in the Supplementary Material illustrates the scatter plots of the gene expression in relation to the post-conceptual age. For instance, the TGFBR1 expression in the amygdala was downregulated in utero (Supplementary Fig. S2a). The IL17RB expression in the hippocampus was downregulated in utero (Supplementary Fig. S2b).

### Cytokine genetic variants modulate the associations between prenatal maternal depressive symptoms and neonatal brain morphology in the GUSTO sample

**Fig. 3:**
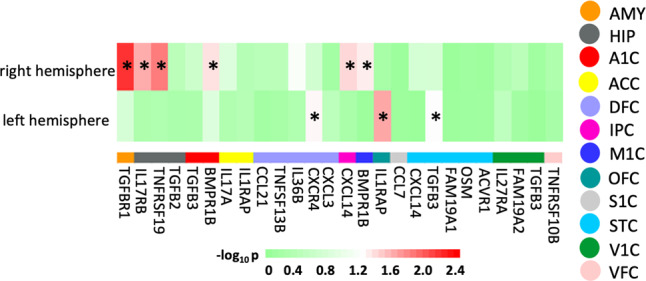
The heatmap for the significant interactions of the cytokine genetic variants with prenatal maternal depressive symptoms on the neonatal brain morphology. The color of solid circles indicates the anatomical annotation of brain regions. *Denotes the statistical significance of the interaction between the cytokine genetic variants and prenatal maternal depressive symptoms on the neonatal brain morphology. AMY amygdala, HIP hippocampus, A1C primary auditory cortex, ACC anterior cingulate cortex, DFC dorsolateral frontal cortex, IPC inferior parietal cortex, ITC inferolateral temporal cortex, M1C primary motor cortex, OFC orbitofrontal cortex, S1C primary sensory cortex, STC superior temporal cortex, V1C primary visual cortex, VFC ventrolateral frontal cortex.

Table 1 lists the mean and standard deviation of the amygdala and hippocampal volumes and cortical thickness of the neonatal brain. We only examined the cytokine genes expressed in the corresponding fetal brain region as shown in Fig. 2. The heatmap in Fig. 3 shows the significant interactions between the genetic variants of the cytokine expressed in specific brain regions and prenatal maternal depressive symptoms upon the brain morphology at birth. The genetic variants of chemokine family and receptors (CXCR4, CXCL14) interacted with prenatal maternal depressive symptoms on the neonatal thickness of the left dorsolateral frontal cortex (DFC; *p* = 0.046) and right inferior parietal cortex (IPC; *p* = 0.032). The genetic variants of TNFRSF19 (*p* = 0.010) and IL17RB (*p* = 0.019) moderate the association between the prenatal maternal depressive symptoms and the right neonatal hippocampal volume. Furthermore, the genetic variants of the IL-1 and TGF family and receptors moderated the relation of prenatal maternal depressive symptoms with the morphology of the emotional and sensory circuits, such as the left orbitofrontal (OFC) thickness (IL1RAP; *p* = 0.017), right amygdala volume (TGFBR1; *p* = 0.004), right auditory (BMPR1B; *p* = 0.036) and motor thickness (BMPR1B; *p* = 0.041), and left superior temporal (STC) thickness (TGFB3; *p* = 0.050). The genetic variants of the remaining cytokine genes in Fig. 2 did not significantly moderate the association between prenatal maternal depressive symptoms and the morphology of the corresponding neonatal brain region (see Table S2 in the Supplementary Material).

**Table 1:** Demographics and brain measures.

Measure	Neonatal sample (*n* = 161)	
	Mean (SD)	Range
Gestational age (week)	38.9 (1.1)	35.9–41.3
Birth weight (gram)	3087.1 (372.1)	2203.6–4001.8
APGAR score	≥9	9–10
Sex, male/female	87/74	
Age at the MRI scan (weeks)	1.41 (0.52)	0.73–2.45
Prenatal maternal depression	8.56 (4.50)	0–21
Maternal ethnicity	%	
Chinese	45.34	
Malay	41.62	
Indian	13.04	
Maternal education	%	
Primary school	3.73	
Secondary school	33.54	
Pre-university, diploma or technical course	44.71	
University undergraduate level	14.29	
Above university undergraduate level	3.73	

Brain region	Mean (SD)	
Total brain volume (cm^3^)	548.71 (43.49)	
Left amygdala volume (mm^3^)	211.13 (34.60)	
Right amygdala volume (mm^3^)	186.57 (33.83)	
Left hippocampus volume (mm^3^)	779.07 (107.11)	
Right hippocampus volume (mm^3^)	783.87 (111.16)	
Left A1C thickness (mm)	2.665 (0.297)	
Right A1C thickness (mm)	2.603 (0.281)	
Left ACC thickness (mm)	2.176 (0.31)	
Right ACC thickness (mm)	1.704 (0.37)	
Left DFC thickness (mm)	2.473 (0.301)	
Right DFC thickness (mm)	2.573 (0.248)	
Left IPC thickness (mm)	2.517 (0.277)	
Right IPC thickness (mm)	2.718 (0.295)	
Left ITC thickness (mm)	2.36 (0.265)	
Right ITC thickness (mm)	2.589 (0.26)	
Left M1C thickness (mm)	2.428 (0.205)	
Right M1C thickness (mm)	2.493 (0.216)	
Left OFC thickness (mm)	2.632 (0.267)	
Right OFC thickness (mm)	2.432 (0.221)	
Left S1C thickness (mm)	2.662 (0.256)	
Right S1C thickness (mm)	2.844 (0.297)	
Left STC thickness (mm)	2.664 (0.277)	
Right STC thickness (mm)	2.635 (0.285)	
Left V1C thickness (mm)	3.407 (0.475)	
Right V1C thickness (mm)	3.014 (0.374)	
Left VFC thickness (mm)	2.732 (0.289)	
Right VFC thickness (mm)	2.741 (0.289)

**Fig. 4:**
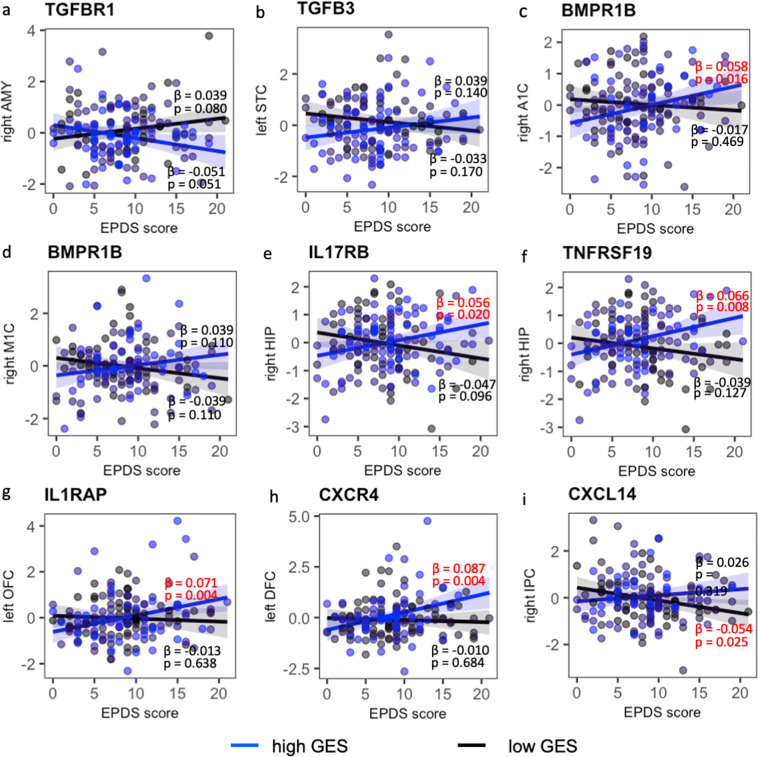
Post hoc analysis of the interaction between prenatal maternal depression and genetic risk on neonatal thickness for the cortex and volume for the amygdala and hippocampus. *Y* axis represents the standardized brain morphology score. The black and blue lines/dots represent the groups with low and high genetic expression scores [39], respectively. The *β* and *p* values in red indicate statistical significance. AMY amygdala, HIP hippocampus, A1C primary auditory cortex, ACC anterior cingulate cortex, DFC dorsolateral frontal cortex, IPC inferior parietal cortex, ITC inferolateral temporal cortex, M1C primary motor cortex, OFC orbitofrontal cortex, S1C primary sensory cortex, STC superior temporal cortex, V1C primary visual cortex, VFC ventrolateral frontal cortex.

Post hoc analysis revealed that in neonates with a high GES of the TGF family and receptors, greater prenatal maternal depressive symptoms were associated to greater thickness of the right auditory cortex (BMPR1B; *p* = 0.016; Fig. 4c). In neonates with a high GES of IL17RB (*p* = 0.02) or TNFRSF19 (*p* = 0.008), greater prenatal maternal depressive symptoms were associated with a larger right hippocampal volume (Fig. 4e, f). Neonates with a high GES of IL1RAP and CXCR4 showed positive associations of prenatal maternal depressive symptoms with left OFC (*p* = 0.004; Fig. 4g) and DFC thickness (*p* = 0.004; Fig. 4h), respectively. Neonates with a low GES of CXCL14 showed a negative association between prenatal maternal depressive symptoms and right IPC thickness (*p* = 0.025; Fig. 4i). These findings suggested the positive associations of prenatal maternal depressive symptoms with the hippocampal volume, auditory and prefrontal cortical thickness in neonates high in GESs of the TNF, IL-1, IL17, chemokine, and TGF family and receptors.

## Discussion

This study used a highly novel approach to integrate the cytokine transcriptome data from the post-mortem fetal brain tissue and neonatal genotype data with neonatal brain images. Our findings showed the upregulation or downregulation of the cytokine gene expressions in utero, suggesting that this small set of the cytokine genes might potentially be functional in a specific brain region in utero. We demonstrated the role of this set of the cytokines in modulating the relationship of the prenatal environment and the accelerated fetal development of the hippocampus, auditory and parietal cortex, orbital, and dorsal prefrontal cortex in utero. In particular, neonates high in the GES of cytokines showed a larger right hippocampal volume (TNFRSF19 and IL17RB), and thicker thickness of sensorimotor (BMPR1B), dorsal and orbital prefrontal cortex (IL1RAP and CXCR4) in relation with greater prenatal maternal depressive symptoms. Our findings suggested that in humans, the cytokine and chemokine genes are expressed in specific fetal brain regions in utero and may have potential roles in modulating the in utero development of the corresponding brain regions in response to the maternal environment.

Recent human studies suggest the accelerated development of specific brain regions as a function of exposure to an adverse maternal environment. This idea is supported by the findings from observational human studies on the amygdala and prefrontal cortex in terms of their larger volumes and/or greater anatomical and functional connectivity in relation with greater prenatal maternal depressive symptoms [5, 7, 40–42]. Prenatal exposure to higher levels of maternal cortisol and depression is associated with child emotional reactivity [40]. Prenatal exposure to maternal cortisol is associated with a larger amygdala volume in 7-year-old children [43]. Greater prenatal maternal depressive symptoms are associated with greater development of the white matter tracts connecting the amygdala and inferior frontal region (i.e., uncinate fasciculus) in 2.6–5.1-year-old children [7]. Similarly, fetal exposure to maternal depression is related to greater left amygdala-left insula functional connectivity in 6-month-old infants [5], which is consistent with the amygdala-insula structural coupling in neonates [9]. A higher amygdala-insula functional connectivity in neonates is found in relation with greater fear in 6-month-old infants [41] and internalizing symptoms at 2 years of age [42]. The findings from this study further support the idea of the brain accelerated development in the context of the maternal environment. This is not restricted to the amygdala and frontal cortex. Our study suggests that such an acceleration model could be applied to in utero development and the other brain regions, such as the hippocampus, sensorimotor, and parietal cortex.

The brain is accelerating development in early life, which has also been discovered in the brain structure and function under the broad context of the maternal environment. For example, infants born to mothers with greater postpartum anxiety have a greater growth of the hippocampus in the first 6 months of life [33]. The early developmental emergence of the prefrontal and amygdala connectivity is observed in children with a history of maternal deprivation [44, 45]. Early life stress, such as maternal post-traumatic stress disorder (PTSD) and a history of early maternal abuse, is associated with an increase in infant cortisol levels [46]. These findings support an idea of life history theory wherein the timing of key developmental achievements is altered by exposure to early adversity [7]. In other words, early childhood demarcates as a critical and malleable period when the brain develops prematurely to adapt to environmental influences in early life.

From the same sample as that in this study, we show that the fetal development of the same brain regions, notably the right hippocampus and left orbitofrontal cortex, is accelerated in relation with a high level of prenatal maternal depression, which is modulated by the genetic variants (e.g., glutamate receptors) with a risk for major depressive disorder [47]. Our findings in this study further demonstrate the role of cytokines in modulating prenatal environment and accelerated left orbitofrontal cortex and hippocampus during in utero development. These findings provide new insights indicating that such accelerated in utero brain development is subject to the individual immune-related genetic susceptibility.

The cytokine genes found in this study are consistent with those highlighted in animal research [48]. In infant rats, maternal deprivation increases microglial activation and neuroinflammatory markers in the prefrontal cortex and hippocampus [48]. Maternally deprived rats show increased interleukin and tumor necrosis factor (TNF-α) levels in the prefrontal cortex but only the level of TNF-α elevated in the hippocampus of infant rats [48]. The TNFRSF19/TROY gene encodes a type I cell surface receptor that is expressed on migrating or proliferating progenitor cells of the hippocampus. The upregulated IL17RB gene is related to the Th2 inflammatory response predominated in the hippocampal tissues of the stressed rats. In line with these findings in animals, our study demonstrates that in neonates with a high GES of TNFRSF19 and IL17RB, greater prenatal maternal depressive symptoms were associated with a larger right hippocampal volume. Likewise, IL1RAP encodes a necessary component of the IL-1 receptor complex and its downstream signaling pathway [49]. IL1RAP rs12053868 is associated with a marker of cortical microglial activation. Moreover, bone morphogenetic proteins (BMPs) that signal through a heterodimeric complex of type I (BMPR1A, BMPR1B, etc) may influence interneuron development and the loss of BMP receptor signaling alters interneuron subtype specification in the cortex and spinal cord [50]. Furthermore, impaired CXCR4 signaling is implicated in abnormal development, proliferation, and migration of neural progenitor cells [51], which is suggestive of their essential roles in neurogenesis. Consistent with these findings in animals, this study provides new evidence from human datasets and demonstrates that in neonates with a high GES of cytokines, greater prenatal maternal depressive symptoms were associated with sensorimotor (BMPR1B), dorsal and orbital prefrontal cortical thickness (IL1RAP and CXCR4) at birth. Our findings suggest that in humans, these genes are expressed in specific fetal brain regions during gestation and may have potential roles in altering the in utero development of these brain regions in response to maternal environment.

Besides, patients with depression exhibit increased expression of pro-inflammatory cytokines and their receptors (e.g., IL-1, IL-6, TNF) and increased levels of chemokines in peripheral blood and cerebrospinal fluid (CSF) [52]. Excessive and/or prolonged activation of cytokines in the brain can diminish neurotrophic support, decrease neurogenesis, increase glutamatergic activation, oxidative stress, induction of apoptosis in relevant cell types (e.g., astrocytes and oligodendrocytes), and dysregulation of glial/neuronal interactions and cognitive function, which are thought to be the pathophysiology of depression [53]. Administration of IL-1 receptor antagonist (IL-1RA) or transplantation of IL-1RA secreting neural precursor cells into the hippocampus blocks the brain cytokine activity and hence prevents the effects of acute and chronic stress on behavior, cognition, neurotrophic factors, and neurogenesis [52, 54]. Increased hippocampal concentrations of TNF and IL-1 are associated with decreased hippocampal BDNF expression and its receptor, and reduced hippocampal neurogenesis, which affects fundamental aspects of neuronal integrity including neurogenesis, long-term potentiation, and dendritic sprouting, ultimately affecting learning and memory [55]. Our findings on the TNF modulation of in utero hippocampal development in the context of maternal depression during pregnancy provide new inflammatory markers that can be potentially considered as one of the inflammation-targeted therapies for depression, particularly during pregnancy.

This study has several strengths, including a longitudinal design of a general population, broad control of potential confounders, direct assessment of the neonatal brain, a relatively large sample size for neonatal brain imaging, and advanced genetic analysis that incorporated spatio-temporal information of genes and genetic variants in imaging genetics. However, this study is best considered as exploratory and intended to provide a strategy for the analysis of gene–environment interdependence and to identify functional genes during gestation that might mediate the influence of environmental conditions on fetal brain development using a general population sample. While preliminary, the neonatal neuroimaging dataset in this study is highly unique in its timing of acquisition and number of subjects, it is nevertheless a modest sample size for genomic analyses. Our assessment of maternal depressive symptoms is based on a common screening tool intended to elicit a subjective report of emotional well-being but does not constitute clinical assessment. The brain variations in the offspring are thus best considered as being associated with self-reported depressive symptoms and not to clinical depression, per se. Moreover, depression and anxiety tend to covary highly with each other, which makes it difficult to parse out their independent contributions to neurodevelopmental outcomes. Last not least, our study did not include potential effects of partner support or early life exposure to adversity of mothers on fetal brain development, which may need further investigation.

In conclusion, we present a hypothesis-generating approach to the study of gene × environment interaction that uses gene transcriptome together with genotypes to identify candidate cytokines moderating the influence of environmental conditions on the in utero brain development. Our findings provide new evidence on cytokines in humans modulating the associations of fetal brain development with prenatal maternal depression, suggesting that inflammatory mechanisms have important roles in regulating the susceptibility to nonoptimal brain development in early life. Our findings on the TNF modulation of in utero hippocampal development in the context of maternal depression provide new inflammatory markers that can be potentially considered as one of the inflammation-targeted therapies for maternal depression, particularly during pregnancy.

## Funding and disclosure

This research is supported by the Singapore National Research Foundation under its Translational and Clinical Research (TCR) Flagship Programme and administered by the Singapore Ministry of Health’s National Medical Research Council (NMRC), Singapore- NMRC/TCR/004-NUS/2008; NMRC/TCR/012-NUHS/2014. Additional funding is provided by the Singapore Institute for Clinical Sciences, Agency for Science Technology and Research (A*STAR), Singapore Ministry of Education (Academic research fund tier 1; NUHSRO/2017/052/T1-SRP-Partnership/01), and NUS Institute of Data Science, Singapore. The authors declare no competing interests.

## Supplementary information

Supplementary
