# Supplementary material for: Inflammatory modulation of the associations between prenatal maternal depression and neonatal brain
Source: Neuropsychopharmacology. 2020 Jul 20;46(2):470–7. doi: 10.1038/s41386-020-0774-0 (PMC7852623; doi:10.1038/s41386-020-0774-0)

**Table S1.** 208 cytokine genes and their number of SNPs.

| **Order** | **Category** | **Gene Name** | **Total Imputed SNP Number** |
| --- | --- | --- | --- |
| 1 | IL1 Family and Receptors | IL18 | 76 |
| 2 |  | IL18BP | 35 |
| 3 |  | IL1A | 33 |
| 4 |  | IL1B | 25 |
| 5 |  | IL1F10 | 84 |
| 6 |  | IL1RN | 106 |
| 7 |  | IL36A | 21 |
| 8 |  | IL36B | 186 |
| 9 |  | IL36G | 39 |
| 10 |  | IL36RN | 75 |
| 11 |  | IL37 | 73 |
| 12 |  | IL18R1 | 247 |
| 13 |  | IL18RAP | 237 |
| 14 |  | IL1R1 | 625 |
| 15 |  | IL1R2 | 236 |
| 16 |  | IL1RAP | 694 |
| 17 |  | IL1RL1 | 309 |
| 18 |  | IL1RL2 | 337 |
| 19 |  | SIGIRR | 50 |
| 20 | IL2 Family and Receptors | IL13 | 21 |
| 21 |  | IL15 | 310 |
| 22 |  | IL2 | 17 |
| 23 |  | IL21 | 52 |
| 24 |  | IL4 | 28 |
| 25 |  | IL7 | 315 |
| 26 |  | IL9 | 25 |
| 27 |  | IL15RA | 203 |
| 28 |  | IL2RA | 322 |
| 29 |  | IL2RB | 128 |
| 30 |  | IL4R | 261 |
| 31 |  | IL7R | 160 |
| 32 | IL6 Family and Receptors | CLCF1 | 44 |
| 33 |  | CSF3 | 24 |
| 34 |  | CTF1 | 15 |
| 35 |  | IL11 | 46 |
| 36 |  | IL31 | 18 |
| 37 |  | IL6 | 47 |
| 38 |  | LEP | 94 |
| 39 |  | LIF | 35 |
| 40 |  | OSM | 28 |
| 41 |  | CNTFR | 193 |
| 42 |  | IL11RA | 37 |
| 43 |  | IL27RA | 108 |
| 44 |  | IL31RA | 272 |
| 45 |  | IL6R | 254 |
| 46 |  | IL6ST | 209 |
| 47 |  | LEPR | 620 |
| 48 |  | LIFR | 533 |
| 49 |  | OSMR | 466 |
| 50 | IL10 Family and Receptors | IL10 | 39 |
| 51 |  | IL19 | 163 |
| 52 |  | IL20 | 12 |
| 53 |  | IL22 | 39 |
| 54 |  | IL24 | 37 |
| 55 |  | IL26 | 120 |
| 56 |  | IL10RA | 94 |
| 57 |  | IL10RB | 162 |
| 58 |  | IL20RA | 269 |
| 59 |  | IL20RB | 143 |
| 60 |  | IL22RA1 | 118 |
| 61 |  | IL22RA2 | 168 |
| 62 | IL17 Family and Receptors | IL17A | 30 |
| 63 |  | IL17B | 39 |
| 64 |  | IL17C | 16 |
| 65 |  | IL17D | 89 |
| 66 |  | IL17F | 45 |
| 67 |  | IL25 | 27 |
| 68 |  | IL17RA | 149 |
| 69 |  | IL17RB | 112 |
| 70 |  | IL17RC | 122 |
| 71 |  | IL17RD | 330 |
| 72 |  | IL17RE | 91 |
| 73 |  | IL17REL | 96 |
| 74 | TNF Family and Receptors | CD70 | 35 |
| 75 |  | FASLG | 26 |
| 76 |  | LTA | 30 |
| 77 |  | LTB | 21 |
| 78 |  | TNF | 28 |
| 79 |  | TNFSF10 | 115 |
| 80 |  | TNFSF11 | 182 |
| 81 |  | TNFSF13B | 130 |
| 82 |  | TNFSF14 | 52 |
| 83 |  | TNFSF15 | 99 |
| 84 |  | TNFSF18 | 45 |
| 85 |  | TNFSF4 | 106 |
| 86 |  | TNFSF8 | 190 |
| 87 |  | TNFSF9 | 33 |
| 88 |  | CD27 | 37 |
| 89 |  | CD40 | 65 |
| 90 |  | EDAR | 396 |
| 91 |  | FAS | 154 |
| 92 |  | LTBR | 66 |
| 93 |  | NGFR | 95 |
| 94 |  | PGLYRP1 | 29 |
| 95 |  | RELT | 96 |
| 96 |  | TNFRSF10A | 126 |
| 97 |  | TNFRSF10B | 245 |
| 98 |  | TNFRSF10C | 115 |
| 99 |  | TNFRSF10D | 415 |
| 100 |  | TNFRSF11A | 349 |
| 101 |  | TNFRSF11B | 167 |
| 102 |  | TNFRSF12A | 19 |
| 103 |  | TNFRSF13B | 205 |
| 104 |  | TNFRSF13C | 15 |
| 105 |  | TNFRSF17 | 34 |
| 106 |  | TNFRSF18 | 33 |
| 107 |  | TNFRSF19 | 573 |
| 108 |  | TNFRSF1A | 71 |
| 109 |  | TNFRSF1B | 199 |
| 110 |  | TNFRSF21 | 412 |
| 111 |  | TNFRSF25 | 35 |
| 112 |  | TNFRSF4 | 28 |
| 113 |  | TNFRSF8 | 348 |
| 114 |  | TNFRSF9 | 83 |
| 115 | IFN Family and Receptors | IFNA1 | 25 |
| 116 |  | IFNA10 | 33 |
| 117 |  | IFNA17 | 32 |
| 118 |  | IFNA2 | 20 |
| 119 |  | IFNA21 | 26 |
| 120 |  | IFNA4 | 23 |
| 121 |  | IFNA5 | 19 |
| 122 |  | IFNA6 | 14 |
| 123 |  | IFNA7 | 21 |
| 124 |  | IFNA8 | 17 |
| 125 |  | IFNB1 | 20 |
| 126 |  | IFNE | 16 |
| 127 |  | IFNG | 15 |
| 128 |  | IFNK | 28 |
| 129 |  | IFNW1 | 11 |
| 130 |  | IFNAR1 | 162 |
| 131 |  | IFNAR2 | 163 |
| 132 |  | IFNGR1 | 93 |
| 133 |  | IFNGR2 | 138 |
| 134 | TGF beta Family and Receptors | TGFB1 | 66 |
| 135 |  | TGFB2 | 341 |
| 136 |  | TGFB3 | 125 |
| 137 |  | ACVR1 | 469 |
| 138 |  | ACVR1B | 163 |
| 139 |  | ACVR2A | 304 |
| 140 |  | ACVR2B | 153 |
| 141 |  | ACVRL1 | 87 |
| 142 |  | AMHR2 | 33 |
| 143 |  | ATF2 | 355 |
| 144 |  | BMPR1A | 737 |
| 145 |  | BMPR1B | 2135 |
| 146 |  | BMPR2 | 613 |
| 147 |  | ENG | 199 |
| 148 |  | TGFBR1 | 218 |
| 149 |  | TGFBR2 | 435 |
| 150 |  | TGFBR3 | 1002 |
| 151 | Chemokine Family and Receptors | CCL1 | 29 |
| 152 |  | CCL11 | 28 |
| 153 |  | CCL13 | 20 |
| 154 |  | CCL16 | 29 |
| 155 |  | CCL17 | 68 |
| 156 |  | CCL18 | 37 |
| 157 |  | CCL19 | 15 |
| 158 |  | CCL2 | 13 |
| 159 |  | CCL20 | 29 |
| 160 |  | CCL21 | 21 |
| 161 |  | CCL22 | 58 |
| 162 |  | CCL23 | 42 |
| 163 |  | CCL24 | 33 |
| 164 |  | CCL25 | 75 |
| 165 |  | CCL26 | 131 |
| 166 |  | CCL27 | 10 |
| 167 |  | CCL28 | 157 |
| 168 |  | CCL4 | 38 |
| 169 |  | CCL5 | 38 |
| 170 |  | CCL7 | 19 |
| 171 |  | CCL8 | 22 |
| 172 |  | CX3CL1 | 81 |
| 173 |  | CXCL1 | 17 |
| 174 |  | CXCL10 | 13 |
| 175 |  | CXCL11 | 13 |
| 176 |  | CXCL12 | 113 |
| 177 |  | CXCL13 | 456 |
| 178 |  | CXCL14 | 43 |
| 179 |  | CXCL16 | 59 |
| 180 |  | CXCL17 | 67 |
| 181 |  | CXCL2 | 12 |
| 182 |  | CXCL3 | 11 |
| 183 |  | CXCL5 | 22 |
| 184 |  | CXCL6 | 11 |
| 185 |  | CXCL9 | 26 |
| 186 |  | FAM19A1 | 2947 |
| 187 |  | FAM19A2 | 2241 |
| 188 |  | FAM19A3 | 37 |
| 189 |  | FAM19A4 | 1106 |
| 190 |  | FAM19A5 | 1945 |
| 191 |  | PF4 | 18 |
| 192 |  | PPBP | 18 |
| 193 |  | XCL1 | 48 |
| 194 |  | CCR1 | 41 |
| 195 |  | CCR10 | 4 |
| 196 |  | CCR2 | 46 |
| 197 |  | CCR3 | 117 |
| 198 |  | CCR4 | 24 |
| 199 |  | CCR6 | 171 |
| 200 |  | CCR7 | 55 |
| 201 |  | CCR9 | 75 |
| 202 |  | CCRL2 | 30 |
| 203 |  | CX3CR1 | 108 |
| 204 |  | CXCR2 | 58 |
| 205 |  | CXCR4 | 19 |
| 206 |  | CXCR5 | 67 |
| 207 |  | CXCR6 | 24 |
| 208 |  | XCR1 | 33 |

**Table S2.** Statistical p-values for the interactions between cytokine genes and prenatal maternal depressive symptoms on the neonatal brain morphology.

| **Brain Region** | **Gene** | **birth** | |
| --- | --- | --- | --- |
|  |  | **left** | **right** |
| amygdala | TGFBR1 | 0.166 | **0.004** |
| hippocampus | IL17RB | 0.558 | **0.019** |
|  | TNFRSF19 | 0.532 | **0.010** |
|  | TGFB2 | 0.610 | 0.394 |
| primary auditory cortex | TGFB3 | 0.600 | 0.226 |
|  | BMPR1B | 0.132 | **0.036** |
| anterior cingulate | IL17A | 0.511 | 0.133 |
|  | IL1RAP | 0.433 | 0.449 |
| dorsolateral frontal cortex | CCL21 | 0.761 | 0.395 |
|  | TNFSF13B | 0.693 | 0.386 |
|  | IL36B | 0.534 | 0.058 |
|  | CXCR4 | **0.046** | 0.196 |
|  | CXCL3 | 0.361 | 0.755 |
| inferior parietal cortex | CXCL14 | 0.632 | **0.032** |
| primary motor cortex | BMPR1B | 0.909 | **0.041** |
| orbitofrontal cortex | IL1RAP | **0.017** | 0.693 |
| primary sensory cortex | CCL7 | 0.908 | 0.981 |
| superior temporal cortex | CXCL14 | 0.991 | 0.176 |
|  | TGFB3 | **0.050** | 0.169 |
|  | FAM19A1 | 0.904 | 0.989 |
|  | OSM | 0.881 | 0.848 |
|  | ACVR1 | 0.848 | 0.915 |
| primary visual cortex | IL27RA | 0.348 | 0.506 |
|  | FAM19A2 | 0.788 | 0.387 |
|  | TGFB3 | 0.995 | 0.837 |
| ventrolateral frontal cortex | TNFRSF10B | 0.678 | 0.555 |

**Figure S1.** Flow chart for the GUSTO sample selection.

**
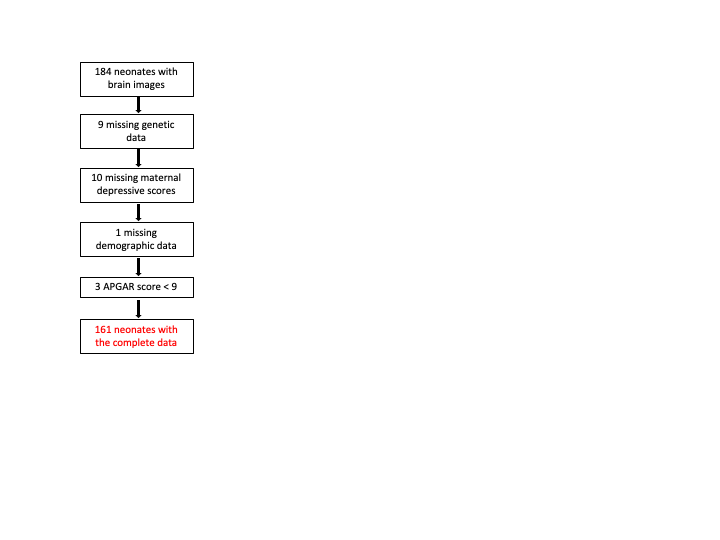
**

**Figure S2.** Scatterplots show gene expression in relation with post-conceptual age. Each panel shows standardized coefficient β-value and its FDR corrected p-value.

Abbreviations: AMY – amygdala; HIP – hippocampus; A1C – primary auditory cortex; ACC – anterior cingulate cortex; DFC – dorsolateral frontal cortex; IPC – inferior parietal cortex; ITC – inferolateral temporal cortex; M1C – primary motor cortex; OFC – orbitofrontal cortex; S1C – primary sensory cortex; STC – superior temporal cortex; V1C – primary visual cortex; VFC – ventrolateral frontal cortex.


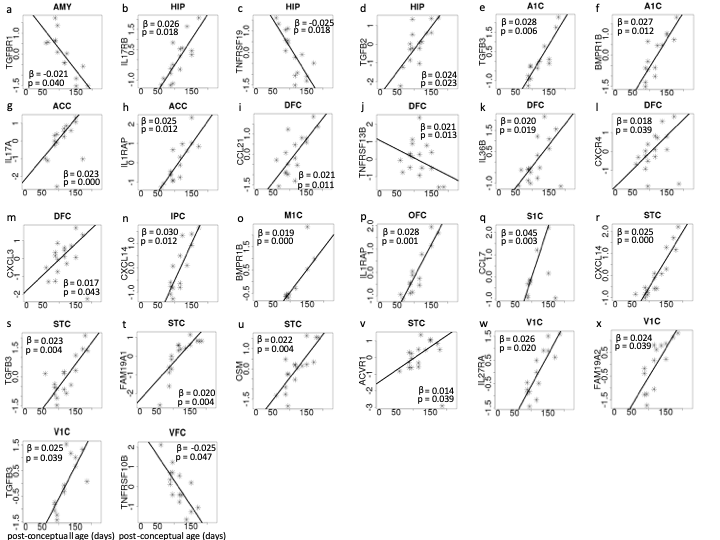

Supplement: Supplementary file 1 — Supplementary [file 41386_2020_774_MOESM1_ESM.docx]
